# Existing public health surveillance systems for mental health in China

**DOI:** 10.1186/1752-4458-9-3

**Published:** 2015-01-05

**Authors:** Wei Zhou, Shuiyuan Xiao

**Affiliations:** Department of Social Medicine and Health Management, School of Public Health, Central South University, 110 Xiangya Road, Changsha, 410078 China

**Keywords:** Public health surveillance, Mental health, China, Review

## Abstract

Mental health is a challenging public health issue worldwide and surveillance is crucial for it. However, mental health surveillance has not been developed until recently in certain developed countries; many other countries, especially developing countries, have poor or even no health information systems. This paper presents surveillance related to mental health in China, a developing country with a large population of patients with mental disorders. Detailed information of seven relevant surveillance systems is introduced respectively. From the perspective of utilization, problems including accessibility, comprehensiveness and data quality are discussed. Suggestions for future development are proposed.

## Introduction

Mental illness is a worldwide challenge in public health. In 2010, mental illness accounted for an estimated 7.4% of the world’s measured in disability-adjusted life years (DALYs), and the years lived with disability (YLDs) resulting from mental and behavioral disorders contributed to 22.7%, the highest YLDs among all diseases [1–3]. Despite the significantly negative impact on people’s life, 35% to 50% of people with severe mental disorders in high-income countries do not receive needed treatment, and the rate can increase to 75% to 85% in low- and middle- income countries [4].

Surveillance is crucial for mental health, as it is the foundation for well-informed and evidence-based decision-making on disease control and prevention, services provision and delivery [5]. WHO’s Comprehensive Mental Health Action Plan 2013–2020 also sets strengthening mental health information system as one of the four objectives [6].

A majority of countries collect data on the number of people treated and service user diagnosis [7]; however, developing systematic surveillance is a recent trend. For example, the collation of existing data as the first step toward developing mental health surveillance was proposed in Canada in 1999 [8]; the Mental health Information and Determinants for the Europe Level Project proposed to develop European mental health information systems in 2006 [9]; for U.S. currently with a relatively mature mental health surveillance network, the government has begun infrastructure construction for establishing an ongoing system for mental health surveillance since 1999 [10]. Despite the short history, mental health surveillance in countries like U.S., U.K., Australia has developed rich content, which can be categorized into four domains: the first is surveillance on mental health problems, including the diagnosis of mental disorders and a range of manifestation of alteration in thinking, mood, behavior and associated with distress that correspond with clinical disorder; the second category is risk factors, like stressors, social support, social and economic status, and birth defects; the third and forth categories are about the mental health services and rights protection for the patients. The surveillance data of the above countries are widely used in government decision-making and academic research [11–15].

Unlike the above countries which develop mental health surveillance on well-developed public health surveillance, many other countries, especially developing countries, have poor or even no health information systems, let alone mental health information systems [7]. This paper will focus on the case of China, a large developing country where there are estimated 16 million patients with psychotic disorders, based on a recent epidemiological survey conducted in four provinces [16]. Existing problems of Chinese surveillance related to mental health are discussed from the perspective of data utilization and suggestions for future development are proposed accordingly.

## Surveillance systems and surveys relevant to mental health in China

After the founding of the People’s Republic of China in 1949, the government first established the Notifiable Infectious Disease Reporting System as a start of disease surveillance. After decades of development, disease surveillance has gradually extended to be public health surveillance with a wider scope, covering not only infectious diseases, but also some non-communicable diseases and health-related factors and events [17]. In 2012, the newly-passed Mental Health Law for the first time explicitly stipulated that a mental health surveillance network should be established (article 24) and that mental health work plans should be based on surveillance results (article 60).

Although China has a basic knowledge of mental health prevalence and mental health services through epidemiological surveys, the National System of Basic Information Collection and Analysis for Psychoses is the only mental-health-themed surveillance system in China. Certain data on mental health are collected through other public health surveillance systems (Table 1) organized by government departments like centers for disease control and prevention (CDCs), the Ministry of Health (MOH [now the National Health and Family Planning Commission]) or provincial departments of health, and statistical departments. Brief introductions for each system are listed as follows.

**Table 1:** **Public health surveillance systems that collect data on mental health**

Category	Name and main sponsor	Mental health topics	Data release
Mental-health-themed surveillance	National System of Basic Information Collection and Analysis for Psychoses, China CDC	Diagnosis of mental illnesses, family history of mental disorders, medication	No data release by so far
Public health surveillance collecting data on mental health	BRFSS, China CDC	Health status, health care and services, tobacco use, alcohol consumption	No regular data releasing
	National Injury Surveillance System, China CDC	Activity when injury happening; cause of injury (including intentional), nature and severity of injury	Detailed statistics published through book series: *Dataset of National Injury Hospital Surveillance (2007–2012)*
	MOH-VR, China MOH	Facts and causes of the death (including suicide)	Data released within health administrative departments
	NDSP-MR, China CDC	Facts and causes of the death (including suicide)	Detailed statistics published through book series: *Dataset of Mortality Registration in NDSP (2004–2011)*
	Working System for National Health Statistics, China MOH	Mental health resources (e.g. the numbers of institutions, beds and staff), service utilization	*Yearbook of China Health Statistics* published annually
	Civil Affairs Statistical Information Management System, China Ministry of Civil Affairs	Mental health services within civil affairs system	Annual reports (1986–2012) available at http://cws.mca.gov.cn/article/tjbg/

### National system of basic information collection and analysis for psychoses

In December 2004, China initiated the National Continuing Management and Intervention Programme for Psychoses (also named 686 Programme), as a response to the Chinese government’s concern on social harmony and stability [18]. One of the program tasks was to register patients diagnosed with 4 types of psychoses, which are schizophrenia, bipolar disorder, delusional disorder, and schizoaffective disorder. One urban and one rural area in each of the 30 provinces in China were selected as pilot sites for this program, and a population of 43 million was covered. By June 2011, the program had developed and been expanded to cover a population of 330 million in 680 urban districts/counties of 160 cities [18, 19].

In August 2011, the online system titled National System of Basic Information Collection and Analysis for Psychoses was released nationwide (http://1.202.129.170:90/mh/). The system operated by CDC, has extended the reporting scope to 6 types of severe mental illnesses: schizophrenia, schizoaffective disorder, bipolar disorder, delusional disorder, psychotic disorder due to epilepsy and mental retardation. Local medical units across China, including mental hospitals, general hospitals with mental health units, community health centers and village clinics, are responsible for case reporting. The system mainly collects 4 categories of information: 1) demographics of patients; 2) management information, including ID number, guardian contacts, informed consent on management; 3) information related to disease and care, including family history of mental disorders, diagnosis, the time for the first disease attack, medication; 4) other information, including financial situation, troubles/accidents conducted and assessment on the risk of violence [20].

### Behavioral Risk Factor Surveillance System (BRFSS)

The system was established in 1996 based on a World Bank project in China, and now China CDC is responsible for its operation. The system targets on urban residents aged from 16 to 69 and collects data through monthly household surveys. It covers about 60 districts and counties located in 7 cities and 1 province and a population of 25 million [17]. Surveys consist of two parts: 1) core questions designed by China CDC and asked in all surveillance districts; 2) questions added by individual cities. 12 sections are included in core questions; they are demographics, health status, health care and services, tobacco use, alcohol consumption, awareness of controlling high blood pressure, awareness of controlling hyperlipidemia, exercise (physical activity), healthy diet, maternal health, accidental injury, knowledge and actions of sexually transmitted diseases (STD) and AIDS. In 1996, approximately 4800 interviews were completed in every city/province as baseline. From 2007 to 2001, there were approximately 4800 completed interviews per year in every city/province [21].

### National injury surveillance system

The system has been operated by China CDC since January 2006. It is sentinel surveillance based on hospital emergency departments [22]. The system samples 43 counties/cities/districts as surveillance sites across China, with 23 in rural areas and 20 in cities. 127 hospitals are included in the system. 3 categories of information are collected through paper reporting cards filled by hospitals: 1) general information of the patient, including name, age, education level, occupation; 2) general information of the injury, including the time, place and reason of the injury, the activity when the injury occurring, intentional or not, and the time of seeing a doctor; 3) clinical information of the injury, including the nature and part of the injury, severity, and clinical diagnosis and outcome. In 2010, the annual number of reporting cards exceeded 620,000 [23].

### Mortality registration and surveillance

Conventional mortality registration and surveillance in China is fulfilled through the Vital Registration System administrated by MOH (MOH-VR) and Mortality Registration in the National Disease Surveillance Point (NDSP) System operated by CDC (NDSP-MR) [24].

MOH-VR was established in 1987. The standard reporting document is a death certificate issued by a hospital, which contains personal information of the dead, the fact (e.g. death sites) and cause of the death (e.g. diagnosis of the fatal disease). In 2004, online reporting from medical institutions at county or above level across China was employed and the Vital Registration Information System was set under China Information System for Disease Control and Prevention (http://www.cdpc.chinacdc.cn/). This promoted the increase of reported deaths by year. In 2006, over 930,000 deaths were reported via the online system, accounting for 12.83% of the total deaths in China. By 2007, the online reporting has covered nearly 80% counties/city districts [25].

The NDSP System sets surveillance points according to a multistage cluster probability sampling with stratification at three levels [24]. By 2006, the system has developed 161 surveillance points and covered over 73 million population [26]. Initially, the NDSP system was designed to collect data on births, causes of death, and the incidence of infectious diseases. Since 1990, the system has covered mortality of 35 notifiable diseases and gradually expanded to deaths of other causes [24]. Similar to MOH-VR, NDSP-MR also depends on death certificates for information collection [27].

### Working system for national health statistics

The primary tasks of the system include: 1) collecting information regarding the input, allocation and utilization of health resources, the quality and efficiency of health services, and the health status of the population; 2) reporting statistical data and analysis; 3) providing statistical consulting and supervision. The whole-process work is administrated by MOH. Medical units at all levels, including metal health institutions, are required to report to local administrative departments of health their health data, including the number of hospital beds and medical staff, the income and expenditure, the person-time of service utilization, and institutions’ ownership [28]. In 2007, the online reporting system for national health statistics was put in use (http://tjbb.zjwst.gov.cn:8080/irpt/i/oem/wsb/login.jsp).

### Civil affairs statistical information management system

In order to monitor the work progress of civil affairs, the Ministry of Civil Affairs established the Work System of Civil Affairs Statistics in 1987. The current information system is functioned with data collection, reporting and analysis. The system can provide data regarding services for the intellectual disability and mental illnesses administrated by departments of civil affairs, including the number of institutions, beds and persons being served [29].

### Other surveillance systems potentially relevant to mental health

U.S. CDC categorizes infectious disease within mental health information [30] and some infectious diseases, like Japanese encephalitis, meningococcal meningitis, rabies, HIV/AIDS and some STDs, can damage the neurologic system or may lead to complications or sequel of mental disorders. Certain birth defects (e.g. meningitis, mongolian idiocy, Down’s syndrome and defects of neurologic-system damage), maternal deaths and causes, social support in early childhood are related to mental health. Therefore, the Infectious Disease Reporting System and the Specific Infectious Disease Management Information System both within China Information System for Disease Control and Prevention, the Information Reporting System of HIV/AIDS, and the Maternal and Child Health Surveillance, also possibly provide information on mental health.

## Problems to be addressed

As ultimate purposes of surveillance are to inform government decision-making and to facilitate academic research, problems of the aforementioned surveillance are proposed mainly from the perspective of utilization.

### Accessibility of data

The aforementioned surveillance systems are all established and operated by government departments and their management documents explicitly state that data are provided as evidence for health administration and decision making [22, 25, 27]. However, information sharing within the health sector and between departments is limited. Longitudinally, the authorization on data access of surveillance organizations is decided by their administrative levels [20]. MOH and China CDC have full access to data across China. A municipal CDC can only view the data of its own city, rather than the whole database of its province. Horizontally, information sharing between different surveillance systems is constrained by the incompatibility of their independent software and hardware [31]. The internal division of duties in the surveillance organizations further reinforces the information blocking. For example, in CDCs, the work related to mental health is included in the division of chronic diseases, which is independent from the division of infectious diseases. As a result, though some infectious diseases are the risk factors of mental disorders, their surveillance are not listed and used in mental health surveillance.

As is presented in Table 1, no data release of BRFSS, MOH-VR, and National System of Basic Information Collection and Analysis for Psychoses have been found by so far, which account for over 1/3 of the listed systems. For the rest ones, summary reports at national level are released to the public, but in variable details and at different time intervals. For example, injury surveillance, NDSP-MR and Working System for National Health Statistics publish detailed statistics of each item on the reporting form, as well as the name list of surveillance points, relevant work manuals, procedures and standards, and government documents in books. Main findings of the Civil Affairs Statistical Information Management System are released through a summary form by year or annual reports.

Even though, it is inconvenient for the academic circle to obtain raw data without summary and analysis/statistical processing. Papers based on raw data of the above systems were mainly published by local surveillance staff using data of their regions [32–34].

### Comprehensiveness of data scope

Compared with the summarized categorization of surveillance content worldwide, surveillance on rights protection is still missing in China, partly because the Mental Health Law passed just a short time ago and some details of implementation are under discussion.

The surveillance categories of mental health problems are limited. First, The National System of Basic Information Collection and Analysis for Psychoses currently only focuses on six severe mental illnesses and other systems only collect data on suicides, intentional injuries, tobacco use and alcohol consumption. Mental disorders commonly found in populations, like depression and anxiety, are not covered and surveillance on substance-related and addictive disorders should go beyond tobacco or alcohol consumption. Second, China mainly focuses on the diagnosed mental disorders and behaviors conducted. However, symptoms of psychological distress without diagnosis, emotional problems (like depression and anxiety) at the screening points, and suicide ideation have not included yet. Third, many surveillance systems in China collect information through self-designed reporting forms, rather than screening instruments, like World Health Organization Disability Assessment Schedule (WHODAS) and AUDADIS-IV (Alcohol Use Disorder and Associated Disabilities Interview Schedule-IV) [11]. The limited number of items in the reporting forms leads to the incomprehensiveness.

In terms of risk factors, unlike US, Europe and Australia including factors like stress, poverty and inequality and poor education in mental health surveillance [12, 14, 35], current surveillance in China mainly includes the biological ones, like damages of neurological system and genetic diseases. Even though surveillance systems containing these biological risk factors, like the Maternal and Child Health Surveillance and the Infectious Disease Surveillance, have not been widely accepted as and used for mental health surveillance in China.

### Quality of data

Quality-control procedures are designed for most surveillance systems. The frequently-adopted methods include self-check, cross check between organizations at the same level and regular check from upper-level organization on the timeliness, completeness and accuracy of data and automatic logical correction by reporting systems [20, 24].

In attempt to check the results of quality control for each of the discussed surveillance systems, we identify evaluation studies on completeness, accuracy and underreporting through the following procedures. We adopted search combinations of “names of surveillance systems” and “quality”, and retrieved academic papers through abstract search in China National Knowledge Infrastructure in August 2014, which has over 90% of China knowledge resources with the widest in title and type coverage and the deepest in year coverage in China. One quantitative evaluation paper was identified for each surveillance under the principles that the studies at national level are selected prior to the ones at provincial or lower levels and that the most recently published studies are selected when there are more than one studies at the same level.

As Table 2 shows that surveillance systems have very limited or even no evaluation studies, which can be partly attributed to the restriction on data access. According to the identified studies, many surveillance systems are underperformed, even only judging from the completeness and accuracy of data, as well as their underreporting rates (Table 2). Factors including the lack of professionals, inadequate training for data reporters, and poor hardware for online reporting in certain areas are proposed as explanations [31, 36]. Repeated surveillance from different systems on certain themes, like the mortality surveillance [31], increases the workload of first-line medical staff and data collectors, which is also negative for surveillance quality.

**Table 2:** **Evaluation studies on data quality of discussed surveillance systems**

Category	Surveillance systems	Number of the retrieved	Identified articles	Evaluation indicators		
				Underreporting rate	Completeness rate	Accuracy rate
Mental-health-themed surveillance	National System of Basic Information Collection and Analysis for Psychoses	1	Reference [37] (City)	--	83.0%	52.8%
Public health surveillance collecting data on mental health	BRFSS	5	--	--	--	--
	National Injury Surveillance System	37	Reference [38] (City)	10.65%	89.89%	--
						--
	MOH-VR	20	Reference [39] (Provincial)	72.67%	--	91.41%
	NDSP-MR	81	Reference [40] (Provincial)	14.44%	85.11%	-
	Working System for National Health Statistics	17	--	--	--	--
	Civil Affairs Statistical Information Management System	0	--	--	--	--

## Suggestions for future development

For the future development of mental health surveillance, a comprehensive surveillance with a full coverage of mental-health-related events is suggested. On the one hand, surveillance indicators should be enriched through expert consultancy and adopting international screening instruments. On the other hand, surveillance scope should be extended to include more categories of mental health problems like depression and substance-related and addictive disorder. Multiple sources of risk factors and law implementation should also be covered. Streamlining the surveillance systems discussed will be a helpful way of expanding surveillance scope.

Second, the government must make the data more accessible for clinical practices, research and evidence-based decision making at all government levels. Publishing surveillance results regularly should become a routine for all surveillance systems. Application procedures for accessing data should be operable. Provisions on privacy protection should also be developed and applied equally to all individual or department data users.

Third, adequate resources for surveillance and implemented evaluations mechanism are keys to quality improvement. To guarantee quality and appropriate collection of data, adequate inputs of funding and staff with necessary training should be provided as a precondition. Systematic and scientific evaluations under published criteria and frameworks should be conducted and guide continuous quality-improvement measures.

## Conclusion

The existing public health surveillance networks lay a good foundation for its future development. Problems in the aspects of data accessibility, comprehensiveness of data scope and quality of data should be solved for better utilization of surveillance in both government decision making and academic research. Promoted by the requirement by Mental Health Law and devoted efforts for improvement, mental health surveillance will be well developed in China.

## Abbreviations

BRFSS: Behavioral risk factor surveillance system
CDC: Center for disease control and prevention
MOH: Ministry of health
MOH-VR: Vital registration system administrated by MOH
NDSP System: National disease surveillance point system
NDSP-MR: Mortality registration in the NDSP System
STD: Sexually transmitted disease.
